# Acquired epileptiform opercular syndrome evaluated with real-time transcranial Doppler ultrasound-video-electroencephalogram before and after treatment: a case report

**DOI:** 10.1186/s12883-019-1389-0

**Published:** 2019-07-17

**Authors:** Bing-Wei Peng, Hui-Ci Liang, Jia-Ling Li, Si-Yuan Yang, Wei Liang, Feng-Qiong Zhang, Wen-Xiong Chen

**Affiliations:** Department of Neurology, Guangzhou Women and Children’s Medical Center, Guangzhou Medical University, 9# Jin Sui Road, 510623, Guangzhou City, Guangdong Province People’s Republic of China

**Keywords:** Acquired epileptiform opercular syndrome, Transcranial Doppler ultrasound, Electroencephalogram, Electrical status epilepticus during sleep (ESES), Children

## Abstract

**Background:**

Acquired epileptiform opercular syndrome (AEOS) with electrical status epilepticus during sleep (ESES) may be recurrent and intractable. The real-time transcranial Doppler ultrasound–sleep-deprived video electroencephalogram (TCD-SDvEEG) can be used to observe the relationships among hemodynamic, electrophysiological, and clinical factors in a patient during therapy. This study reported the case of a healthy 5-year-old boy with AEOS.

**Case presentation:**

The patient had initial seizures during sleep at the age of 1 year, with the left mouth pouting, left eye blinking and drooling for several seconds, and, sometimes, the left upper-limb flexion and head version to the left, lasting for 1–2 min. The combined antiepileptic drug regimens, including valproate, lamotrigine, and clonazepam, failed in the present case. Therefore, the add-on high-dose methylprednisolone therapy was provided. Also, the serial TCD-SDvEEG was used to monitor the dynamic changes before and after add-on steroid treatment. The results showed less than 15% variation in the range of blood flow fluctuation with spikes during non-rapid eye movement sleep after treatment. This was similar to the outcomes in healthy children and also accorded with the clinical improvements such as seizure control, drooling control, and language ability melioration. However, 95% of spike-wave index (SWI) was still maintained. The improvements in cerebral hemodynamics and clinical manifestations were faster and earlier than the SWI progression.

**Conclusions:**

The real-time TCD-SDvEEG was highly sensitive in detecting therapeutic changes. The findings might facilitate the understanding of the mechanisms underlying neurovascular coupling in patients with AEOS accompanied by ESES.

## Introduction

The acquired epileptiform opercular syndrome (AEOS) is a rare epileptic syndrome, which manifests with typically focal motor seizures, involving the face, and occasionally rolandic seizures, accompanied by severe oral motor dysfunction, drooling, dysarthria, speech arrest, or linguistic problems involving phonologic productions [1]. Electrical status epilepticus during sleep (ESES) may appear when the spike wave index (SWI) in sleep is 85% or greater [2]. AEOS with ESES may be recurrent and intractable. The conventional electroencephalogram (EEG) may not be sensitive and timely enough to detect the changes in treatment.

Recently, the notion of tight coupling between cerebral blood flow velocity (CBFV) and brain activity has been widely accepted. Transcranial Doppler ultrasound–sleep-deprived video electroencephalogram (TCD-vEEG) is a promising technique proving neurovascular coupling [3]. A previous study [4] showed changes in cerebral hemodynamics during the non-rapid eye movement sleep in different stages in healthy children using TCD-sleep-deprived vEEG (TCD-SDvEEG). The total effectiveness rate of steroids on ESES suppression in benign childhood epilepsy with centro-temporal spikes (BECT) variants with ESES was found to be 82% [5]. This study aimed to report the case of a pediatric patient with AEOS receiving add-on high-dose intravenous steroid therapy. The therapy seemed to be partially effective only on the clinical conditions but did not affect ESES suppression. The serial real-time TCD-SDvEEG was then carried out to dynamically observe the changes in the relationships among hemodynamic, electrophysiological, and clinical factors before and after steroid therapy. Written informed consent was obtained from the parents.

## Case presentation

A previously healthy 5-year-old boy was admitted to the hospital. He was a full-term baby delivered via vaginal sections following a normal 39-week pregnancy with unremarkable events. He did not require support services after birth and had no family history of epilepsy. The child had normal development, including expressive and receptive language, at the appropriate time.

This patient had initial seizures during sleep at the age of 1 year, with the left mouth pouting, left eye blinking and drooling for several seconds, and, sometimes, left upper-limb flexion and head version to the left, lasting for 1–2 min. Electroencephalography (EEG) showed rolandic cortex discharges, with 40% SWI. The regimens of valproate 0.25 bid, lamotrigine 25 mg bid, and clonazepam 0.5 mg bid were administered, and consequently, the frequency of seizures reduced.

However, in August 2016, clinical deterioration with extra manifestations, including continuous drooling, dysarthria, and expressive language impairments, occurred, in accordance with the ESES presentation of EEG (i.e., SWI > 85%). As a result, steroids [methylprednisolone (MPN): 10 mg/(kg · d) for 3 days, followed by 5 mg/(kg · d) for 3 days] and intravenous immunoglobulin [IVIG; 1 g/(kg · d) for 2 days], followed by oral prednisone (starting with the initial dose of 2 mg/kg, lasting for 1 or 3 months, and then tailed off) were provided to the patient in the later serial sessions, with the same antiepileptic drugs regimen as earlier. He was relieved with no seizure and drooling. Improvement in language ability was observed, but the patient had a recurrence after prednisone was tailed off every 4–5 months.

**Fig. 1:**
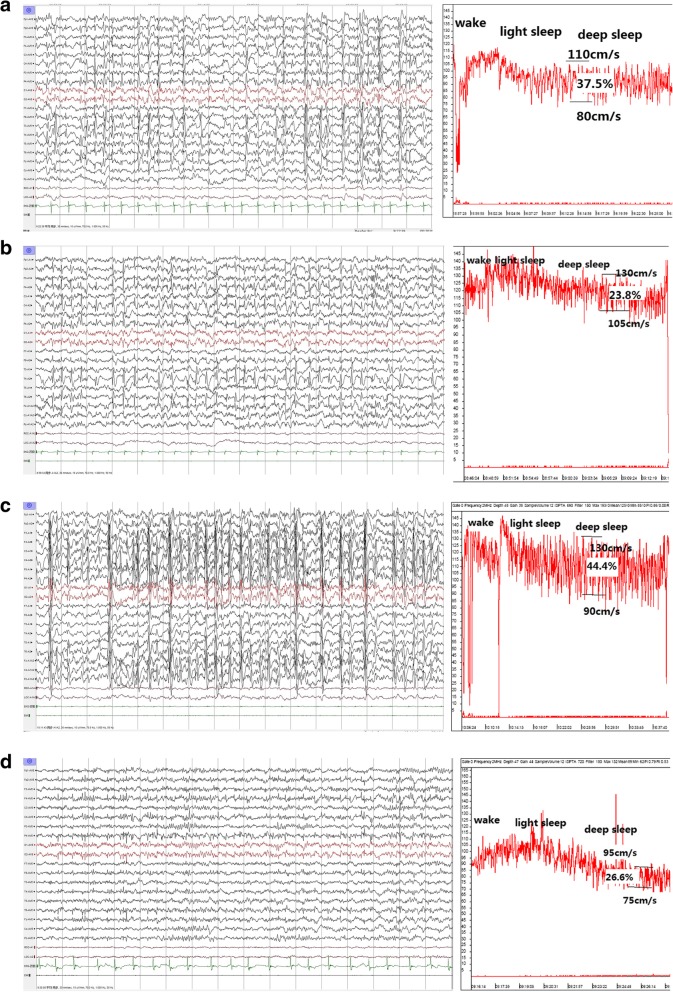
(**a**–**d**) The serial real-time transcranial Doppler ultrasound–sleep-deprived video electroencephalogram (TCD-SDvEEG). *F* (fluctuation of CBFV) = peak CBFVm – baseline CBFVm)/baseline CBFVm. (**a**) August 2017, after the seizure, drooling and dysarthria were recurrent. Involuntary emotional movements of the face were apparent, besides decreased speech output with moderate dysarthria. EEG: ESES, 95% SWI. TCD tracings had no change during NREM sleep, *F* > 35% (37.5%). (**b**) After IVIG (1 g/kg for 2 days) and high-dose methylprednisolone therapy [10 mg/(kg · d) for 3 days, followed by 5 mg/(kg · d) for 3 days], the seizure was controlled, with no drooling and fluency in the language as earlier. EEG: ESES, obviously on the right side, 90% SWI. TCD tracing from light sleep to deep sleep was similar to that in healthy children, *F* < 25% (23.8%). (**c**) The patient had frequent seizures with left eye blinking, drooling, and slightly unfluent language 1 week after prednisone withdrawal. EEG: ESES, 95% SWI. TCD tracings had no change during NREM sleep, *F* > 35% (44%). (**d**) After 3-day high-dose MPN course of 10 mg/(kg · d), followed by a 3-day course of 5 mg/(kg · d), and then oral prednisolone 2 mg/(kg · d) for a month, no seizure was observed, and cognition and language showed continuous improvement. EEG: 40% SWI. TCD tracing from light sleep to deep sleep was similar to that in healthy children, *F* < 30% (26.6%)

Therefore, since August 2017, the serial real-time TCD-SDvEEG monitoring was carried out. The outcomes of the stable transcranial Doppler ultrasound (TCD) tracings and the corresponding EEGs in the following serial relief-and-relapse sessions are shown in Fig. 1a–d.

Specifically, in August 2017, the patient experienced similar recurrent types of seizures, drooling, and dysarthria, with involuntary movements of the face for about 10 days (Fig. 1a). The real-time serial TCD-vEEG monitoring was then applied as previously described [4]. The trends in lack of CBFV variation and obvious range of CBFV fluctuation (more than 35%) were found during all non-rapid eye movement (NREM) sleep stages (Fig. 1a; TCD), in accordance with the corresponding EEG with 95% SWI (Fig. 1a; EEG). Interestingly, after administering steroids and IVIG, the outcomes of TCD tracing (Fig. 1b; TCD) returned to nearly normal levels [4], consistent with the resolution of clinical symptoms (Fig. 1b). Moreover, the range of CBFV fluctuation also alleviated with the changes in spikes, despite no changes in SWI (95%) (Fig. 1b; EEG). Unfortunately, 1 week after prednisone withdrawal (October 09, 2017), the symptoms relapsed, in keeping with the deterioration of TCD tracing (Fig. 1c; TCD), although the ESES status still showed no suppression compared with the last session (Fig. 1c; EEG). Because of repeated recurrence, the long-term steroid regimen was provided with methylprednisolone [10 mg/(kg · d) for 3 days, followed by 5 mg/(kg · d) for 3 days], followed by the long-term oral prednisone maintenance.

Specifically, after 1 month of oral administration of prednisone at a dose of 2 mg/kg, the improvements in clinical symptoms (Fig. 1d), decrease in SWI (40%) (Fig. 1d; EEG), and parallel changes in hemodynamics (Fig. 1d; TCD) were observed.

## Discussion and conclusions

A child with prolonged but intermittent drooling, lingual dyspraxia, and electroencephalographic (EEG) features compatible with benign childhood epilepsy with centrotemporal spikes was described by Roulet et al. in 1989 [6]. Soon after, two cases with dysarthria, dysphagia, and hypersalivation accompanied by ESES were stated as AEOS in 1995 [1]. Thus, the present case was diagnosed as AEOS according to the clinical characteristics, including partial seizure in sleep and paroxysmal oro-facio-lingual deficits with ESES and no other abnormal conditions [7]. Shafrir and Prensky speculated that AEOS and Landau-Kleffner syndrome (LKS) might share a similar pathophysiological mechanism in which long-standing electrical dysfunction of perisylvian neurons caused bilateral neurological dysfunction [1]. The present case had a primary epileptic focus in the right Sylvian fissure (Fig. 1b and d**; EEG**), most likely anatomically located in the lower rolandic cortex and operculum insulae. Consequently, the operculum syndrome occurred with a deterioration in EEG generalization to the bilateral (Fig. 1a and c**; EEG**) operculum insulae.

Valproate and lamotrigine in polytherapy are often effective in AEOS. Also, clobazam might affect AEOS. However, refractory ESES to antiepileptic drugs, including valproate, benzodiazepines, and lamotrigine, often requires high-dose steroid or adrenocorticotropic hormone therapy [8]. The clinical and electrographic improvements are often transient, as in the recurrent case. Steroid and intravenous immunoglobulin (IVIG) have been used for treating LKS. In the present case, add-on high-dose intravenous steroids and IVIG were effective too. However, the course of steroid therapy is still debated [9]. In the present case, short-time high-dose steroid therapy was effective but relapse was possible. During the last recurrent session, long-term steroid therapy regimen was provided, which successfully led to ESES suppression (Fig. 1d).

The pathophysiological mechanism underlying AEOS is unclear. Brain magnetic resonance imaging (MRI) revealed no structural anomalies in AEOS. The magnetoencephalographic analysis showed broadly distributed epileptic foci around the Sylvian fissure in an AEOS case [10]. A fluorodeoxyglucose positron emission tomography brain scan showed a hypometabolic area in the right mesiotemporal area in one AEOS case [11]. In other cases, single-photon emission computed tomography (SPECT) also showed a localized high-perfusion area [12]. Transcranial Doppler ultrasound–electroencephalogram (TCD-EEG) is a promising technique [13]. Compared with ictal SPECT or functional MRI, it is inexpensive, portable, and advantageous in providing the temporal pattern of neurovascular coupling [14]. A previous study [4] found that healthy children during the NREM sleep showed hemodynamic changes in different stages with increased CBFV in the middle cerebral artery during light sleep (N1 stage) and reduced systolic CBFV in all vascular arteries during deep sleep (N3 stage) using TCD-SDvEEG monitoring technique. Usually, more than 10% variation compared with baseline CBFV in 1 min indicates the presence of abnormal CBFV changes in TCD. The changes in cerebral hemodynamics from light sleep to deep sleep in healthy children can be interpreted as neurovascular coupling.

The clinical improvements and EEG ameliorations of ESES may not be coincident [8]. Hence, it is necessary to seek other more sensitive markers to reflect the clinical response to treatment. Consequently, the monitoring test was used in the present case with intractable AEOS to explore the hemodynamics and brain activity before and after the steroid treatment. The analysis of TCD tracings and EEG findings showed that clinical improvement was consistent with the changes in TCD tracings irrespective of SWI reduction.

Recently, hemodynamic responses were found to contribute to the pathogenesis of epilepsy [15]. The outcomes of serial TCD-SDvEEG proved that the hemodynamic changes were more sensitive than the progression of SWI, in accordance with the clinical improvements in the present case. The results further supported the neurovascular coupling mechanism in AEOS. It was speculated that improvement in blood flow might occur in the early stage of recovery of ESES, while improvement in EEG might lag. However, because of the neurovascular coupling mechanism, the relationship between EEG and clinical is complex.

The cerebral hemodynamic responses have been widely adopted to map brain function in humans [16]. Previous studies proved that seizures were associated with substantially increased cerebral blood flow [13]. However, the effects of interictal epileptiform discharges (IEDs) on brain function are unclear. IEDs and cerebral hemodynamic response have not been investigated earlier. ESES is the continuous IEDs during NREM sleep, which may lead to increased neuronal activity. Therefore, cerebral blood flow (CBF) in patients with ESES during NREM sleep possibly increases to meet normal brain activity. The increased CBF during NREM sleep can be reflected by no downward trend in CBFV and increased CBFV fluctuation during deep sleep.

These novel findings indicated distinct alterations in cerebral hemodynamics using serial real-time TCD-SDvEEG for AEOS before and after the high-dose steroid therapy. Thus, real-time TCD-SDvEEG monitoring is more sensitive than single vEEG and may facilitate an understanding of neurovascular coupling in ESES during sleep. The present study further supported the hypothesis that the epileptic discharges produced clinical symptoms, in accordance with the neurovascular coupling mechanisms [15].

## Data Availability

Not applicable.

## Abbreviations

AEOS: Acquired epileptiform opercular syndrome
BECT: Benign epilepsy of childhood with centrotemporal spikes
CBFV: Cerebral blood flow velocity
EEG: Electroencephalogram
ESES: Electrical status epilepticus during sleep
IEDs: Interictal epileptiform discharges
IVIG: Intravenous immunoglobulin
LKS: Landau-Kleffner syndrome
MPN: Methylprednisolone
MRI: Magnetic resonance images
NREM: Non-rapid eye-movement
SPECT: Single photon emission computed tomography
SWI: Spike wave index
TCD: Transcranial Doppler ultrasound
TCD-EEG: Transcranial Doppler ultrasound -video-electroencephalogram
TCD-SDvEEG: Transcranial Doppler ultrasound - sleep-deprived video-electroencephalogram
vEEG: video electroencephalogram
